# Treatment with an HDL mimetic peptide resembles the protective Christchurch variant in rescuing APOE4 secretion and lipidation deficits in primary and iPSC‐derived astrocytes

**DOI:** 10.1002/alz70855_102528

**Published:** 2025-12-23

**Authors:** Kristina Fredriksen, Siddhi S. Joshi, Minwoo Kim, Ling Li

**Affiliations:** ^1^ University of Minnesota, Minneapolis, MN, USA

## Abstract

**Background:**

Apolipoprotein E (APOE) is the greatest genetic risk factor for Alzheimer's disease (AD) and in humans has 3 isoforms; APOE4 (E4) increases risk, APOE3 (E3) is neutral and APOE2 (E2) decreases risk. APOE is mainly produced and secreted by astrocytes, where it binds lipids to form HDL‐like particles. The lipidation state of APOE is crucial for maintaining lipid homeostasis and regulating Aβ aggregation and protein clearance. E4 is poorly lipidated compared to E2 and E3, contributing to cellular dysfunction. Emerging therapies aim to restore E4 lipidation levels to resemble E2 or E3. We previously showed that the HDL‐mimetic peptide 4F increases APOE secretion and lipidation in primary mouse and human astrocytes. Furthermore, recent evidence suggests that the protective APOE Christchurch (APOEch) mutation alters APOE properties to resemble E2. However, the effects of HDL mimetic peptides and APOEch on APOE4 astrocytes remain unclear. We hypothesize that both pharmacological (4F) and genetic (APOEch) interventions enhance APOE secretion and lipidation, and rescue E4‐related deficits.

**Method:**

We generated APOE4 Christchurch (E4ch) induced pluripotent stem cells (iPSCs) and knock‐in (KI) mice using CRISPR/Cas9. E3, E4 and E4ch isogenic iPSCs were differentiated into astrocytes. Primary astrocytes were isolated from E4 and E4ch KI mice. Astrocytes were incubated with aggregated Aβ42 and/or the HDL mimetic peptide 4F for 24 hours. Secreted media and cell lysates were subjected to non‐denaturing gradient gel electrophoresis and immunoblot analyses.

**Result:**

Compared to E4, E4ch primary and iPSC astrocytes enhanced APOE secretion and lipidation. Aβ42 inhibited APOE secretion in E4 astrocytes. Importantly, E4ch astrocytes were more resilient, maintaining elevated APOE secretion and lipidation levels in the presence of Aβ42. E4ch astrocytes also promoted lysosomal protein clearance more than E4 astrocytes. Remarkably, 4F treatment counteracted the inhibitory effects of Aβ42 and enhanced APOE secretion and lipidation in both primary and iPSC astrocytes.

**Conclusion:**

HDL mimetic peptide 4F treatment resembles the protective effects of E4ch and mitigates E4‐related deficits on APOE secretion and lipidation in astrocytes. These findings highlight the potential of targeting APOE secretion and lipidation as a therapeutic strategy for sporadic AD.

